# Theoretical Calculation and Simulation of Peak Distortion of Absorption Spectra of Complex Mixtures

**DOI:** 10.1177/00037028241297179

**Published:** 2024-12-10

**Authors:** Rui Cheng, Thomas G. Mayerhöfer, Johannes Kiefer

**Affiliations:** 1 Technische Thermodynamik, University of Bremen, Bremen, Germany; 2 Spectroscopy and Imaging, Leibniz Institute of Photonic Technology (IPHT), Jena, Germany; 3 Institute of Physical Chemistry and Abbe Center of Photonics, Friedrich Schiller University, Jena, Germany; 4 MAPEX Center for Materials and Processes, University of Bremen, Bremen, Germany

**Keywords:** Peak distortion, attenuated total reflection spectroscopy, ATR spectroscopy, Kramers–Kronig transform, solvent infrared spectroscopy

## Abstract

Attenuated total reflection (ATR) spectroscopy in infrared is a standard tool used in most analytical labs, as it allows a rapid chemical analysis with virtually no sample preparation. However, when the sample contains materials with a high refractive index, special care must be taken as the resulting data may be severely biased. This article reports a theoretical approach to correcting distorted ATR spectra. Starting from Snell's law, Lorenz model and Fresnel's equations are combined to obtain the complex relationship between optical constants. With calculating the real and imaginary parts, that is, n(ν) and k(ν) , respectively, of the complex refractive index from the absorption spectrum, a model for mixtures comprising of a liquid and a solid is established. The effects of distortion and potential misinterpretation of the data are discussed. Proof-of-concept experiments with mixtures of carbonaceous materials and toluene confirm the theoretically predicted observations.

## Graphical abstract

**Graphical abstract:**
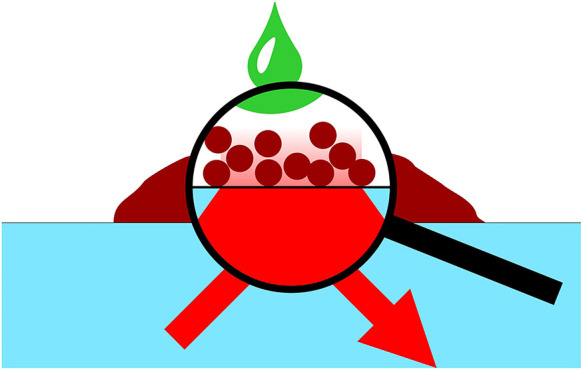
This is a visual representation of the abstract.

## Introduction

Attenuated total reflection (ATR) techniques are widely used in spectroscopy due to their experimental simplicity and non-destructive nature.^1–7^ In ATR, radiation is propagating in a material with high refractive index, often referred to as the internal reflection element (IRE), and undergoes total internal reflection at the IRE surface. This surface is in contact with the sample such that the evanescent field^8^ of the radiation can interact with the sample. In ATR absorption methods, internally reflected radiation carries information about the absorption spectrum of the sample.

The most widely used range for the ATR technique is the mid-infrared spectral range^9^ (i.e., from ∼4000 to ∼200 cm^–1^). The aim is usually to either carry out a structural analysis from assigning the peaks to vibrational modes or to perform quantitative measurements (e.g., as a means of process analytical technology). However, there are a number of issues arising from complex, chemically, and physically inhomogeneous samples and extracting reliable information from their spectra. This is particularly true for strongly absorbing samples and media with a high refractive index, such as carbon black,^10,11^ coal,^12^ graphite, graphene, rubber,^13,14^ metal,^15–17^ metal oxide,^18^ etc.^19^ When such samples are analyzed, the ATR spectrum is always affected by significant distortions.^20–23^ As those distortions can originate from a multitude of physical and chemical phenomena, it is vitally important to understand the underlying mechanisms and to develop methods for their correction. Most of these issues are related to the fact that the refractive index is so high that the critical angle is higher than the one used in the ATR accessory; hence, the total internal reflection is affected.

In the present work, we focus on the analysis of mixtures of particles mixed with a solvent as suggested for the recently developed solvent infrared spectroscopy approach, which can be used to study the surface chemistry of nanomaterials.^24^ Such systems are particularly interesting as they seem to be simple at first glance because the IRE–solvent interface appears to be dominating the evanescent field. However, we will show that the reality is more complicated. For this purpose, a theoretical framework based on Snell's law and the Kramers–Kronig (KK) relationship^25,26^ has been developed. From this framework, a method for correcting distortions in experimental spectra is proposed. Simulations and experimental data are presented to illustrate the phenomena. We note that the model itself is of general nature and can be used to treat classical cases of samples as well.

## Experimental

### Materials and Methods

Two different carbonaceous materials were used: (i) Carbon black powder from Aladdin Reagent Database Inc., and (ii) activated carbon pellets that are commonly used for air purification and solvent recovery (CarboTech C40/4 Extra). Toluene was purchased from Sigma-Aldrich, Inc. All materials were used as received without further purification.

### Experimental Measurements

Infrared measurements were conducted on an Agilent Cary 630 Fourier transform infrared (FT-IR) spectrometer (Agilent Technologies Inc., USA) equipped with a diamond ATR sampling accessory (single reflection at 45°). The full range accessible with the instrument (i.e., 650–4000 cm^−1^) was recorded with a nominal resolution of 2 cm^−1^. A total of 32 scans were averaged to achieve an optimal signal-to-noise ratio. The measurements were carried out at ambient temperature (*T* ≈ 295 K) and pressure (*p* ≈ 1013 mbar).

Each measurement protocol entailed an initial deposition of the solid sample onto the crystal and applied pressure with the instrument's metal stamp to ensure full contact between the solid sample and the IRE. Subsequently, toluene was added.

## Theoretical Considerations

### Snell's Law and Attenuated Total Reflection Spectroscopy

As aforesaid, in ATR, the sample is in contact with a high refractive index material in which radiation undergoes total internal reflection. For clarity and to define a suitable starting point for further theoretical considerations, we briefly introduce the physical basics that describe this situation.

When light propagates from one transparent medium into another, Snell's law applies at the interface:^27^

(1) $$n_{1}\sin\;\theta_{1}=n_{2}\sin\;\theta_{2}$$

where *n*_1_ and *n*_2_ are the refractive indices of the two transparent media, and θ_1_ and θ_2_ are the angle of incidence and refraction with respect to the normal interface, respectively. The critical angle of total reflection is given as

(2) $$\theta_{c}=\sin^{-1}\frac{n_{2}}{n_{1}}$$

In turn, a critical refractive index can be defined for $\theta_{2}=90\circ$ as follows:

(3) $$n_{c}=\sin\theta_{1}n_{1}$$

If the refractive index of the second material, *n*_2_, remains below this value, radiation will be totally reflected inside material 1. This is desirable in ATR, as the sample should only interact with the evanescent field. The situation becomes challenging if the refractive index of the sample is close to the critical refractive index.

Most commercially available ATR units are operated at an angle of incidence of 45° or 60°, and the typical materials are diamond, germanium, and zinc selenide. Table I gives an overview of the resulting critical refractive indices using the IRE refractive index at 10 µm (= 1000 cm^–1^).

**Table I.** Refractive index and critical sample refractive indices for common IRE materials for common angles of incidence. Note that only two digits are given as this precision translates to what can approximately be achieved in adjusting the angle in an experiment.

IRE material	*n* at 1000 cm^–1^	45°	60°
Diamond	2.38^28^	1.69	2.06
Ge	4.00^29^	2.83	3.47
ZnSe	2.40^30^	1.70	2.08

For most inorganic and organic substances, the refractive index is well below 1.7. Even if the refractive index is higher than the critical value in some parts of the spectrum (e.g., due to absorption signatures), the resulting ATR data will be informative, but spectral interpretation will be complicated by band shape changes and peak position shifts. If the refractive index rises above the critical value in a narrow range around a spectral signature, the ATR technique is still useable. However, some substances exhibit a high refractive index and strong absorption transitions in the entire spectral range of interest. For instance, when carbonaceous materials and related products, such as rubber, are to be analyzed, even IRE materials with a high refractive index like Ge are not completely satisfying. When such materials are analyzed by ATR spectroscopy, there will be peak shifts, complicated baselines, and complex and abnormally shaped peaks. These phenomena can lead to misinterpretation (e.g., when the peak shifts are attributed to molecular interactions).

### Kramers–Kronig Relationship

In order to develop a better understanding, we need to consider the complex refractive index, $\hat{n}(\nu )$ :

(4) $$\hat{n}(\nu )=n(\nu )+ik(\nu )$$

with n(ν) and k(ν) being the real and imagine parts, respectively, and ν being the frequency. The real part n(ν) in Eq. 4 is the quantity typically referred to as the refractive index (see for example the values in Table I). The imaginary part k(ν) in Eq. 4 is related with the attenuation of the field amplitude as the electromagnetic wave travels through the material.^31^ In absence of other attenuation effects, the imaginary part is equal to the absorption coefficient. In infrared spectroscopy, the wavenumber (typically expressed in reciprocal centimeters, cm^–1^) is commonly used as abscissa in spectral plots. Consequently, when representing the refractive index in the context of infrared spectroscopy, it is standard practice to use wavenumber instead of frequency. This convention arises because the wavenumber is directly proportional to energy and inversely proportional to wavelength, providing a more intuitive scale for analyzing vibrational and rotational transitions in molecular spectroscopy. Because in ATR, when the incident angle is greater than the critical angle ( $\theta_{c}$ ), or the sample refractive index is below the critical refractive index ( $n_{c}$ ), no “real” light, but the evanescent wave, enters the sample in non-absorbing regions. Absorption leads to the corresponding attenuated total reflection spectrum. For an accurate treatment of refraction and absorption, the complex refractive index needs to be taken into consideration.

The relationship between $\hat{n}(\nu )$ , n(ν),k(ν) , and the relative dielectric constant $\epsilon_{r}(\nu )$ is frequently studied. For instance, Mayerhöfer lays them out and explains them in the context of ATR spectroscopy in a series of papers.^31–36^ Therefore, we reproduce only the main equations here and refer the reader to the original papers for further details. The relative dielectric constant is a complex number:

(5) $$\epsilon_{r}(\nu )=\epsilon_{r}^{\prime }(\nu )+i\epsilon_{r}^{\prime \prime }(\nu )$$

with $\epsilon_{r}^{\prime }(\nu )$ and $\epsilon_{r}^{\prime \prime }(\nu )$ being the real and imaginary parts, respectively. Assuming that the oscillator parameters (oscillator strength *S*, center frequency $\nu_{0}$ , and the damping constant γ ) in Lorenz's model are known, the related parameters of the material,^11^ including $\varepsilon_{r}(\nu )$ , $\hat{n}(\nu )$ , n(ν) , and k(ν) , can be determined in a straightforward manner (for simplicity, we only assume a single oscillator):

(6) $$\epsilon_{r}(\nu )=1+\frac{S^{2}}{\nu_{0}^{2}-\nu^{2}-i\nu \gamma }$$

(7) $$\hat{n}(\nu )=\sqrt{\epsilon_{r}(\nu )}$$

(8) $$n(\nu )=\mathrm{real}(\hat{n}(\nu ))$$

(9) $$k(\nu )=\mathrm{imag}(\hat{n}(\nu ))$$

The parameters n(ν) and k(ν) are connected through the KK relationship:^37^

(10) $$n(\nu )=n_{\infty }+\frac{2}{\pi }P\int_{0}^{\infty }\frac{\nu^{\prime }k(\nu^{\prime })}{\nu {{}^{\prime }}^{2}-\nu^{2}}{d\nu }^{\prime }$$

(11) $$k(\nu )=-\frac{2\nu }{\pi }P\int_{0}^{\infty }\frac{n(\nu^{\prime })}{\nu {{}^{\prime }}^{2}-\nu^{2}}d\nu^{\prime }$$

where $n_{\infty }$ is the real part of the refractive index at high frequencies, and *P* is the Cauchy principal value integral. Mathematically, the KK relationship is essentially the Hilbert transform:

(12) $$\hat{n}(\nu )=n(\nu )+ik(\nu )$$

(13) $$n(\nu )=n_{\infty }+\frac{1}{\pi }P\int_{-\infty }^{\infty }\frac{k(\nu^{\prime })}{\nu^{\prime }-\nu }{d\nu }^{\prime }$$

(14) $$k(\nu )=-\frac{1}{\pi }P\int_{-\infty }^{\infty }\frac{n(\nu^{\prime })}{\nu^{\prime }-\nu }d\nu$$

Consequently, the Hilbert transform can be utilized in a numerical calculation. For instance, in Matlab, it does not require an external function and is therefore convenient and fast. In this paper, the Hilbert transform is uniformly adopted to replace the KK transform in calculations. The relationship between the KK transform and the Hilbert transform is shown in Figure S1 (Supplemental Material) for completeness.

### Fresnel's Equations

In order to take polarization effects into account, the Fresnel equations need to be considered in the model. Their derivation from Maxwell's equations can be found in any optics textbook. The final set of equations is given as follows:^38^

(15) $$r_{s}=\frac{n_{1}\cos\theta_{1}-\sqrt{n_{2}^{2}-n_{1}^{2}\sin^{2}\theta_{1}}}{n_{1}\cos\theta_{1}+\sqrt{n_{2}^{2}-n_{1}^{2}\sin^{2}\theta_{1}}\;}$$

(16) $$r_{p}=-\frac{n_{2}^{2}\cos\theta_{1}-n_{1}\sqrt{n_{2}^{2}-n_{1}^{2}\sin^{2}\theta_{1}}}{n_{2}^{2}\cos\theta_{1}+n_{1}\sqrt{n_{2}^{2}-n_{1}^{2}\sin^{2}\theta_{1}}\;}$$

(17) $$R_{S}=|r_{s}{|}^{2}={\left|\frac{n_{1}\cos\theta_{1}-\sqrt{n_{2}^{2}-n_{1}^{2}\sin^{2}\theta_{1}}}{n_{1}\cos\theta_{1}+\sqrt{n_{2}^{2}-n_{1}^{2}\sin^{2}\theta_{1}}\;}\right|}^{2}$$

(18) $$R_{p}=|r_{p}{|}^{2}={\left|\frac{n_{2}^{2}\cos\theta_{1}-n_{1}\sqrt{n_{2}^{2}-n_{1}^{2}\sin^{2}\theta_{1}}}{n_{2}^{2}\cos\theta_{1}+n_{1}\sqrt{n_{2}^{2}-n_{1}^{2}\sin^{2}\theta_{1}}\;}\right|}^{2}$$

(19) $$R=\frac{R_{s}+R_{p}}{2}$$

(20) $$A=-\log_{10}R$$

The parameters $\theta_{1}$ and $\theta_{2}$ are the incident angle and the refraction angle, respectively. Furthermore, $n_{1}$ and $n_{2}$ are the refractive indices of medium 1 and medium 2, respectively, and $r_{s}$ and $r_{p}$ are the complex amplitude coefficients of *s-* and *p-*polarized light, respectively.^39^ The reflectance for *s-* and *p-*polarized light is *R_s_* and *R_p_*, while *R* is their mean value assuming an unpolarized light source. Parameter *A* is eventually the absorbance calculated from the reflectance.

Note that Eq. 19 provides an approximately calculated value when the incident light is naturally polarized (unpolarized). In general, light is not naturally polarized in an ATR accessory since optical elements like mirrors change the polarization state in dependence of the wavenumber. It is possible to take the polarization state into account for correcting ATR spectra, but, in this work, we assume that the light is approximately naturally polarized. In particular, for ATR accessories employing multiple reflections, this approximation may no longer be appropriate, and our formalism would need to be extended.^40,41^

## Simulation Approach

### Obtaining n(*ν*) and k(*ν*) from Absorption or Reflection

In this section, we present the stepwise calculation procedure. Please refer to Figure S2 (Supplemental Material) for a basic flowchart. For illustration purposes, we consider a mixture of carbon and toluene in order to show results based on real data.

First, the values for n(ν) and k(ν) need to be obtained from an absorption or reflection spectrum. The k(ν) value can be derived in a straightforward manner from,^11^

(21) $$A(\nu )=\frac{4\pi d\nu k(\nu )}{\ln10}$$

(22) $$k(\nu )=\frac{\ln10A(\nu )}{4\pi d\nu }$$

where *d* is the path length. The path length as such does not exist in ATR, but an effective path length can be determined from the penetration depth *d*_p_. The amplitude of the evanescent wave decays to 1/*e* of its maximum value at a distance *d*_p_ from the interface. This distance is on the order of the wavelength of light and given by

(23) $$d_{p}=\frac{1}{2\pi \nu \sqrt{n_{1}^{2}\sin^{2}\theta_{1}-n_{2}^{2}}}$$

This parameter can be converted to an effective path length *d*_eff_, which is the path length that would lead to the same absorption in a transmission experiment. The latter assumes low absorption, the same assumption *d*_eff_ is derived from. *d*_eff_ depends on *d*_p_ and the polarization state of light.^42,43^ In order to focus on the relevant math here, we simplify

(24) $$d=2d_{p}=\frac{1}{\pi \nu \sqrt{n_{1}^{2}\sin^{2}\theta_{1}-n_{2}^{2}}}$$

and obtain

(25) $$k(\nu )=\frac{\ln10A(\nu )}{4}\sqrt{n_{1}^{2}\sin^{2}\theta_{1}-n{\left(\nu \right)}^{2}}$$

Furthermore, k(ν) is connected with n(ν) through the Kramers–Kronig relationship. Here, the method to calculate n(ν) and k(ν) from A(ν) is according to Bertie and Satoru Nakashima.^44–46^ It includes the following steps:

Step 1: $n_{\infty }$ is calculated. (The refractive indices of toluene, $n_{\mathrm{To}}$ , and carbon, $n_{C}$ , were taken from Myer et al.^47^ and Sorensen et al.,^48^ respectively.) The calculation of $n_{\infty }$ uses a modified Sellmeier equation:

(26) $$n(\nu )=\sqrt{a+\frac{b}{\lambda^{2}-c}}$$

where *a*, *b*, and *c* are constant numbers that are derived from fitting the equation to experimental data. Note that the *n*(ν) in Eq. 26 is measured at non-absorbing frequencies. Please find a detailed explanation in the supporting information (Figure S3, Supplemental Material). It derives from Eq. 6 under the assumption of zero damping (i.e., without absorption). At this point, *n*(ν) is only used to calculate $n_{\infty }$ . It must not be confused with the final calculation of the *n*(ν) spectrum. The resulting best-fit equation was used to find the refractive index at the highest energy data point in the experimental spectra. For our model system toluene/carbon, we obtain $n_{\mathrm{To}\infty }=n_{\mathrm{To}(4000\;cm^{-1})}\;=1.4707$ and $n_{C\infty }=n_{C(4000\;cm^{-1})}\;=1.79$ .

Step 2: Replace *n* by $n_{\mathrm{To}\infty }$ to calculate k(ν) by Eq. 25. If the refractive index $n_{\infty }$ is too high (i.e., the square root in Eq. 25 becomes negative), then use Eq. 27 instead of Eq. 25 to have a starting value for the iteration process:

(27) $$k_{\;j+1}(\nu )=k_{j}(\nu )(1+\sqrt{k_{j}(\nu ))}$$

in which, $k_{\;j+1}(\nu )$ is the renew value from the old value $k_{j}(\nu )$ .

Step 3: The obtained k(ν) is used to calculate n(ν) by the Hilbert transform in Eq. 13.

Step 4: Get the complex refractive index $\hat{n}(\nu )$ by Eq. 4.

Step 5: Treat $\hat{n}(\nu )$ as $n_{2}$ , and apply Fresnel's equations, which means Eqs. 15–20 to get $A(\nu {)}_{cal}$ .

Step 6: Calculate the new $k_{\;j+1}(\nu )$ by Eq. 28

(28) $$k_{\;j+1}(\nu )=k_{j}(\nu )\frac{A(\nu )}{A{\left(\nu \right)}_{cal}}$$

Repeat Steps 3–5 until

(29) $$\sum (A(\nu )-A{\left(\nu \right)}_{cal}{)}^{2}<10^{-4}$$

Then, stop the calculation to obtain *n*(ν) *and k*(ν).

In the low-wavenumber range, there may exist a non-convergence using the abovementioned approach. This is attributed to strong absorption features and the real part of the refractive index n(ν) changing significantly. In this case, k(ν) from Myer et al.^47^ is applied as Eq. 22 is directly used for the calculation. The fitting method is changed from Eqs. 28 to 30, which we found empirically:

(30) $$k_{\;j+1}(\nu )=k_{j}(\nu )+\frac{A(\nu )}{500A{\left(\nu \right)}_{cal}}$$

Finally, the two pieces of data are spliced to get the complete data set. The measured n(ν) and k(ν) composite refractive index is not used as the experimentally measured $n_{\mathrm{To}\infty }=n_{\mathrm{To}(8000\;cm^{-1})}\;=1.4749$ is far outside the experimental interval studied in this paper. The comparison of n(ν) and k(ν) calculated from the literature and this paper is shown in Figure S4 (Supplemental Material).

As the refractive index of carbon is larger than the critical refractive index, the corresponding complex refractive index cannot be calculated in this way. Therefore, A(ν) is used to replace k(ν) in order to calculate n(ν) . We need to keep in mind that this contributes to the experimental error in the present work. Moreover, the complex refractive index is not calculated by ellipsometric or surface reflective infrared (SRIR) measurements because these instruments cannot provide the same pressure as diamond ATR, which has a great influence on the experimental spectrum.

**Figure 1.**
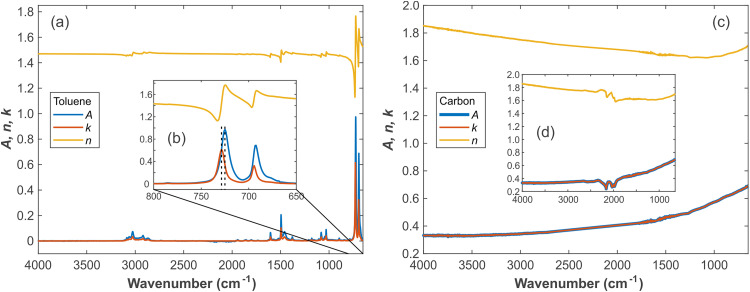
Spectra of pure toluene and carbon. (a) Overview A(ν) , n(ν) , and k(ν) of toluene; (b) zoomed-in low wavenumber region of toluene spectra; (c) overview A(ν) , n(ν) , and k(ν) of carbon with diamond absorption removed; and (d) A(ν) , n(ν) , and k(ν) of carbon with diamond absorption displayed. The dashed lines in (b) indicate the apparent peak shift between the measured absorbance spectrum and the derived k(ν) .

Figure 1 shows the n(ν) and k(ν) values of toluene calculated from A and $n_{\infty }$ . The observed peak of absorbance is significantly redshifted compared to k(ν) (Figure 1b). The k(ν) peak is consistent with the peak position in the transmission infrared spectrum. Although k(ν) and the absorbance spectrum are similar, the positions of their peaks are not the same, so that the absorbance spectrum cannot be used directly. Generally, it must be noted that the measured peak positions in ATR FT-IR, transmission IR, and reflection IR spectra differ and that even for Ge as ATR crystal corrections are needed, although the deviations between k(ν) and the absorbance spectrum are smaller in this case.

The signatures around 1800–2200 cm^–1^ in Figure 1d are caused by two-phonon absorptions of the IRE material diamond.^49,50^ This absorption feature is of great significance for determining whether the distortion of the spectrum is caused from optical reasons, that is, the non-attenuated total reflection caused by the excessive refractive index of the sample. Strictly speaking, the refractive index of diamond should be treated as a complex number, but then we would have to switch to a more complex optical model that treats the ATR crystal as an incoherent layer. However, since most samples have no absorption in this wavenumber region, and diamond does not exhibit absorption signatures elsewhere in the considered spectral range, we treat diamond as a non-absorbing medium here. This assumption helps to keep the complexity of the calculation relatively small. When simulating or correcting the spectrum, this part can be directly replaced with a straight line or corresponding curve to reduce the simulation error.

### Solid/Liquid Mixture Infrared Absorption Spectrum Model

The n(ν) and k(ν) data obtained by the above method are mixed to model and calculate the absorption spectrum in different ways. Medium 2 represents the mixed substances.

(31) $$n_{1}=2.3778$$

(32) $${\hat{n}}_{21}(\nu )=n_{21}(\nu )+ik_{21}(\nu )$$

(33) $${\hat{n}}_{22}(\nu )=n_{22}(\nu )+ik_{22}(\nu )$$

${\hat{n}}_{21}(\nu )$ is the complex refractive index of the liquid, like toluene in our experiment. $n_{21}(\nu )$ and $k_{21}(\nu )$ are real and imaginary parts of ${\hat{n}}_{21}(\nu )$ , respectively. ${\hat{n}}_{22}(\nu )$ is the complex refractive index of the solid, with non-characteristic absorption, but high refractive index, similar to the case for carbon black in the experiment. The refractive index of the mixture is then given as follows:

(34) $${\hat{n}}_{2}(\nu )={\hat{n}}_{21}(\nu )+x{\hat{n}}_{22}(\nu )\;(x=x_{1},x_{2})$$

For completeness, the following intuitive equation in liquid mixing is invalid in this situation:

(35) $${\hat{n}}_{2}(\nu )=(1-x){\hat{n}}_{21}(\nu )+x{\hat{n}}_{22}(\nu )$$

Figure S5 (Supplemental Material) shows the comparison between the spectra obtained from Eqs. 34 and 35 for demonstration purposes.

Liquid mixing does not necessarily follow the same trends as solid–liquid mixing. Eq. 34, which was developed to describe the characteristics of solid–liquid mixtures, might not be applicable to liquid-only mixtures. Solid–liquid mixing often involves complex interactions due to the disparate physical states and properties of the components, such as differences in refractive index, light scattering, and absorption behavior. These factors contribute to spectral distortions unique to solid–liquid mixtures. If *x* would be the mole or volume fraction, Eq. 35 should be (approximately) valid, but since Eq. 34 much better describes the experimental findings, *x* cannot be identified with one or the other. Since carbon black is insoluble in water and other solvents, this may be the reason that Eq. 34 is favorable over Eq. 35.

In the model, the complex refractive index comprises

(36) $$n_{2}(\nu )=\mathrm{real}({\hat{n}}_{2}(\nu ))=n_{21}(\nu )+x_{1}n_{22}(\nu )$$

(37) $$k_{2}(\nu )=\mathrm{imag}({\hat{n}}_{2}(\nu ))=k_{21}(\nu )+x_{2}k_{22}(\nu )$$

**Figure 2.**
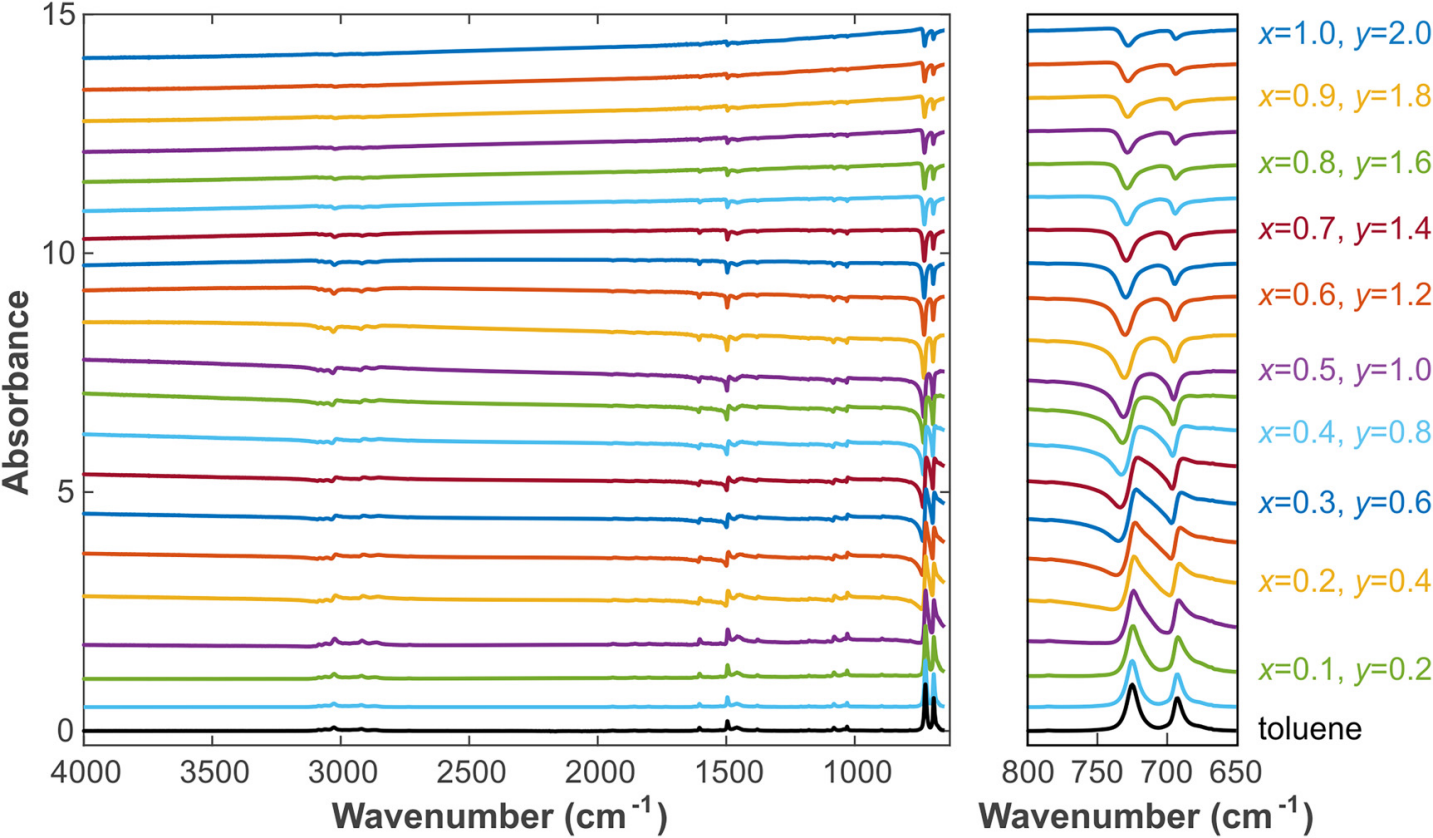
Simulated absorbance spectrum with varying *x* and *y* using Eq. 34 in combination with Fresnel's equations. The initial toluene spectrum is displayed at the bottom. The parameters *x* and *y* are increased in steps of 0.05 and 0.1, respectively. The left panel shows the overall spectrum, and the right panel shows the enlarged region 650–800 cm^–1^. Spectra are displayed with offset for clarity.

We substitute these equations into Fresnel's equations (Eqs. 17–20) to yield the corresponding absorbance *A*, which can then be used to calculate the final simulated absorbance *Ab* (formally both *Ab* and *A* refer to absorbance, but *Ab* is simply used to distinguish the two in different situations):

(38) $$Ab=A+yA_{22}+a\;(0\leq y)$$

where $A_{22}$ corresponds to the absorbance of carbon black in the experiment. The obtained *Ab* spectrum is shown in Figure 2. Figure S6 (Supplemental Material) shows the comparison between *A* and *Ab* for clarity.

Figure 2 shows that with increasing *x*, that is, when the refractive index of the mixture sample gradually rises, the summit first redshifts, and a concave part appears on the left side of the peak (the concave part is difficult to observe in the beginning, so many researchers treat the spectrum as “normal” and regard the redshifted peak as the result of intermolecular interactions). This is caused by the superposition of n(ν) of the complex refractive index. Then, the concave part becomes more and more obvious, and the normal peak becomes weaker until central symmetry is maintained. The same effect can be observed when the incident angle of the radiation is changed: when the incident angle changes from large to small approaching the critical angle, a redshift can be seen, and the left side of the peak begins to appear concave. This was demonstrated by Amma et al.^39,48^ However, for a given absorption transparent medium, the peak shape with one side recessed and the other protruding will continue to be maintained with decreasing incidence angle, and just the degree of inclination will change.

**Figure 3.**
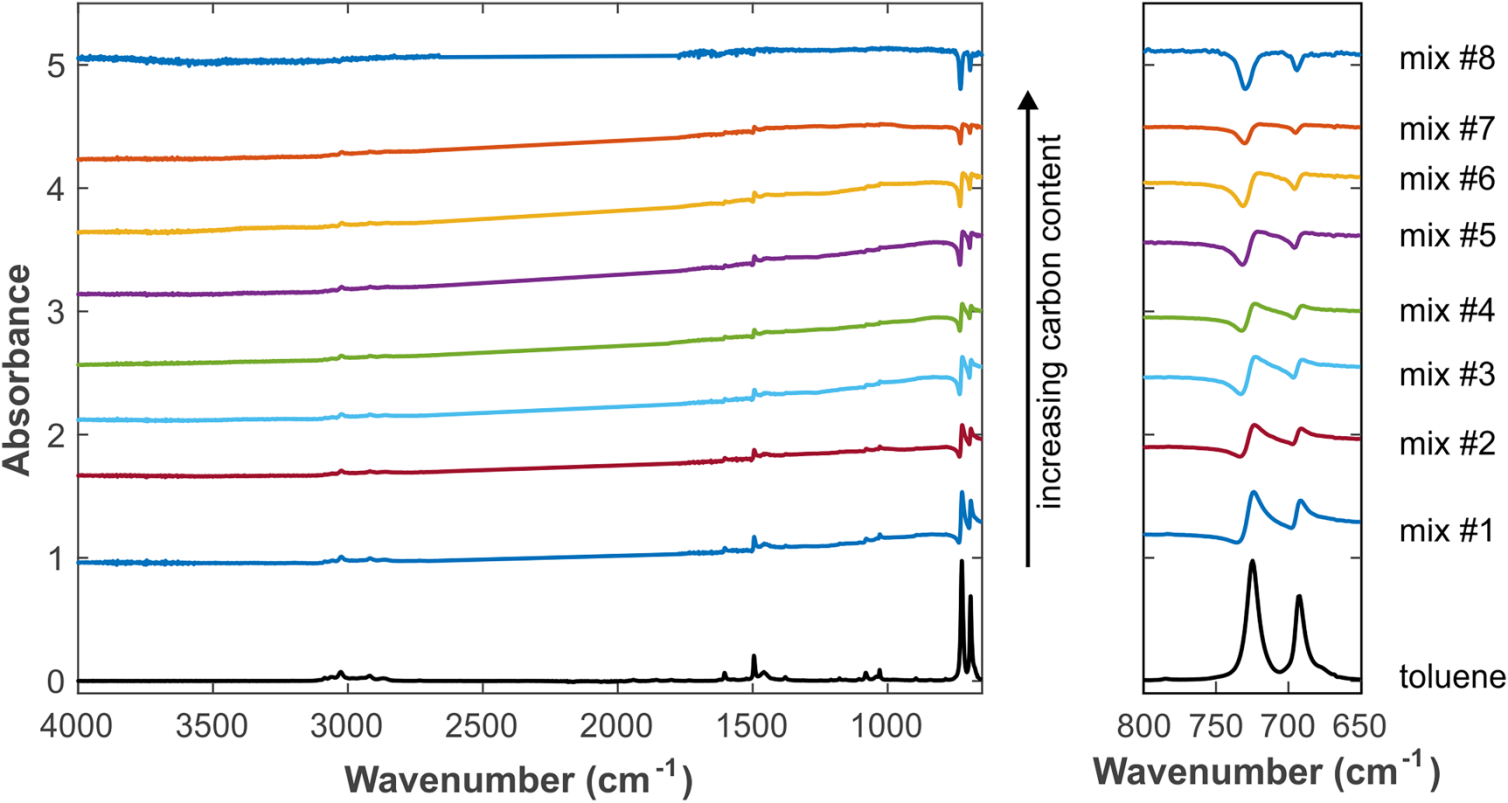
Experimental spectra of different mixtures containing carbon black and toluene. The spectrum of pure toluene is displayed at the bottom. The left panel shows the overall spectra, and the right panel displays the enlarged region 650–800 cm^–1^. Spectra are displayed with offset for clarity.

**Figure 4.**
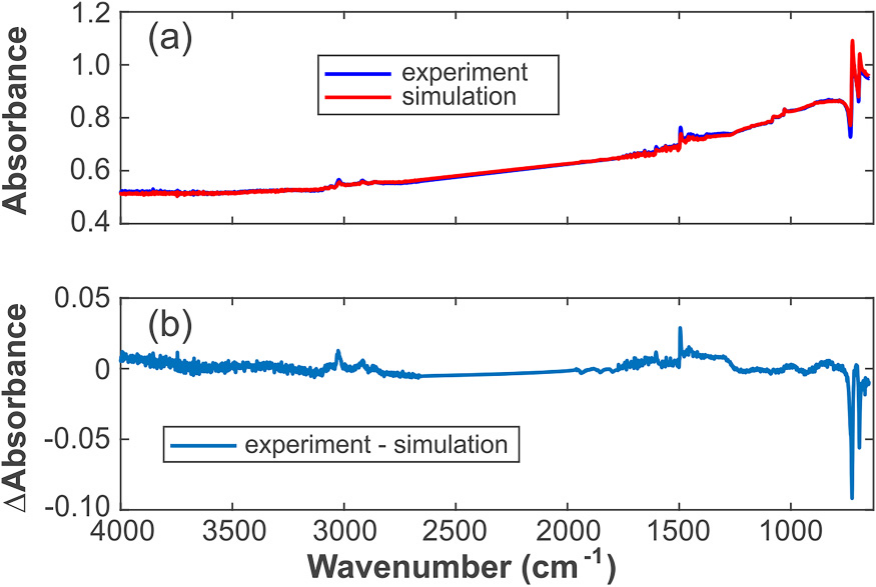
(a) Experimental and simulated spectra of the sample mix no. 3 and (b) their difference spectrum.

Please note that in mixtures with a high refractive index and non-selective absorption, it is different from the selective absorption peak of a transparent medium. After the central symmetry, the peak of the recessed part will be more obvious. The peak from here began to change from center symmetry to the negative peak. Until the original positive peak completely disappeared, the negative peak appeared. The entire absorption spectrum will be overall inverted and show a blueshift. This phenomenon can be verified from the mixed graph of pure carbon black and toluene shown in Figure 3. With increasing *y*, the baseline starts to tilt. In the low wavenumber regime, the baseline starts to rise, which is caused by the absorbance of carbon black, see Figures 1b and 4. However, when it reaches a certain level, the low wavenumber baseline starts to drop again, while the high wavenumber baseline slowly rises until it finally becomes the inverted, blueshifted peak of the original liquid absorption peak. Of course, the increases of *x*_1_, *x*_2_, and *y* are almost synchronized, which shows that *x*_1_, *x*_2_, and *y* are not independent variables.

To summarize the above, when light is refracted by the optically dense medium into the optically thin medium, the distortion of the spectrum caused by the real part n(ν) of the complex refractive index might already occur before the calculated critical angle is reached. In turn, this means that when the refractive index of the sample is less than the critical refractive index, the spectrum is already affected, that is, the peaks are redshifted, and correction is needed to get the accurate peak position. Note that what we call redshift here should precisely be called a pseudo redshift, which is caused by the light itself, not due to the intermolecular interaction or other physicochemical phenomena in the sample. In many cases, this observed redshift is an actual blueshift in the unaffected/corrected spectrum. Therefore, the effect under study can lead to severe misinterpretations of datasets.

When the mixture contains a substance with non-selective absorption, and the refractive index of the sample is high enough, the infrared peaks of the sample can be observed anything from a positive to negative appearance and from redshift to blueshift. This is particularly the case when the mixture contains carbonaceous substances such as carbon black, graphite, or graphene. These substances do not provide distinct absorption peaks in the infrared, so such peaks can be observed only when other organic substances are admixed. Unfortunately, carbon itself can cause the distortion of the baseline; the baseline will rise sharply at low wavenumbers, and because of its high reflectivity, the normal infrared spectrum is almost invisible when the content is higher than 20%. This is even true for Ge-ATR (i.e., under the condition that the refractive index is smaller than the critical refractive index of Ge).

## Comparison of Theory and Experiment

In order to qualitatively test and validate the theoretical model and results presented above, a series of experiments with mixtures of carbon black and toluene were carried out. The exact composition at the IRE–sample interface is difficult to determine, but the experimental procedures were such that a clear variation of the carbon content at the IRE interface could be achieved, which is sufficient for our comparison. Figure 3 shows the spectra obtained with different proportions of carbon black and toluene. The development of the experimental spectra with increasing carbon content agrees well with the theoretical trends revealed in Figure 2. This demonstrates that the model includes all relevant effects.

The calculation and fitting method of the spectrum are a nonlinear fit to determine the parameters *x*_1_, *x*_2_, *y*, and α. The data of the mix no. 3 sample (see Figure 3) are shown in Figure 4, with the removed diamond absorption peaks between 1775 and 2661 cm^–1^. The data and variance are provided in Table S1 (Supplemental Material).

The difference spectrum in Figure 4b shows small deviations from zero, which may be caused by the following: (i) A(ν) was used instead of k(ν) for calculating the complex refractive index of the carbon black; and (ii) only the influence of the compound refractive index of the mixture was taken into consideration as the reason for the distortion. In fact, there are many factors that could influence the result, including the solid carbon content, the solid–liquid ratio, the type of liquid, the shape and size of the solid, the solid–liquid molecular interactions, and a numerical error, among others. As the spectrum simulated using the above model shows a good agreement with the experiment and is capable of predicting the overall trends, we are confident that all relevant phenomena are included.

## Conclusion

The present work aims at better understanding the ATR spectra of complex mixtures (i.e., mixtures of organic or inorganic liquids with solids exhibiting a high refractive index). The ATR spectra of such mixtures are subject to severe distortions that can lead to misinterpretation of the data. We explored the reasons for the change of the spectrum near the critical angle and theoretically simulated the shape change and displacement of absorption peaks. The theoretical results were confirmed in experiments using toluene and carbon black as a model system. Moreover, the mixing law of n(ν) of the complex refractive index and its impact on the resulting spectrum were studied. The proposed model allows the correction of distorted spectra and therefore aids the correct interpretation of experimental results. The model and correction method, however, are not limited to the infrared spectral range. Thus, the method can be applied across the entire spectrum. The development of further specific simulation procedures, elaboration on the curve correction, and analysis of spectral calibration are the subjects of ongoing work.

## Supplemental Material

sj-docx-1-asp-10.1177_00037028241297179 - Supplemental material for Theoretical Calculation and Simulation of Peak Distortion of Absorption Spectra of Complex Mixtures Supplemental material, sj-docx-1-asp-10.1177_00037028241297179 for Theoretical Calculation and Simulation of Peak Distortion of Absorption Spectra of Complex Mixtures by Rui Cheng, Thomas G. Mayerhöfer and Johannes Kiefer in Applied Spectroscopy
