# Supplementary material for: Theoretical Calculation and Simulation of Peak Distortion of Absorption Spectra of Complex Mixtures
Source: Appl Spectrosc. 2024 Dec 10;79(5):852–61. doi: 10.1177/00037028241297179 (PMC12053103; doi:10.1177/00037028241297179)
Supplement: sj-docx-1-asp-10.1177_00037028241297179 - Supplemental material for Theoretical Calculation and Simulation of Peak Distortion of Absorption Spectra of Complex Mixtures [file sj-docx-1-asp-10.1177_00037028241297179.docx]

**Supplemental Material**

**Theoretical Calculation and Simulation of Peak Distortion of Absorption Spectra of Complex Mixtures**

Rui Cheng,^1^ Thomas G. Mayerhöfer,^2,3^ and Johannes Kiefer^1,4*^

^1^Technische Thermodynamik, University of Bremen, Badgasteiner Str. 1, 28359 Bremen, Germany

^2^Leibniz Institute of Photonic Technology (IPHT), Albert-Einstein-Str. 9, 07745 Jena, Germany

^3^Institute of Physical Chemistry and Abbe Center of Photonics, Friedrich Schiller University, Helmholtzweg 4, 07743 Jena, Germany

^4^MAPEX Center for Materials and Processes, University of Bremen, 28359 Bremen, Germany

*Corresponding author email: jkiefer@uni-bremen.de

**Comparison Hilbert and KK Transform**

As an example, Figure S1 shows a comparison and the subtle differences between $n\left( \text{ν} \right)$ and $k\left( v \right)$ calculated for a given set of oscillator parameters ($S=10000\mathrm{cm}^{-1}$,$\text{ν}_{0}=1700\mathrm{cm}^{-1}$, and $\gamma=10 \mathrm{cm}^{-1}$) ^1^ and $k\left( \text{ν} \right)$ calculated from $n\left( \text{ν} \right)$ via the Hilbert and the KK transform. The $k\left( \text{ν} \right)$ calculated by the Hilbert transform shows only minor deviations from the ‘real’ $k\left( \text{ν} \right)$. The error is within an acceptable range and can be neglected in the vicinity of the absorption feature. However, the figure also reveals that if the Hilbert transform or KK transform is directly applied to the spectrogram, more severe distortion will occur with increasing distance from the absorption center line (absorbance peak). So before taking the relevant transformation, the lowest value and the highest value were supplemented with corresponding data at both sides respectively, and the supplementary data was cut off after the transformation. This ensures the accuracy of the data and the integrity of the spectrum. Please note that the two $k\left( \text{ν} \right)$ lines in Fig.S1a, the red solid line is the k obtained by KK transformation, and the yellow dashed line is the $k$ obtained by the Hilbert transformation, agree with each other very well. They cannot be distinguished by the naked eye in the figure. This shows that the Hilbert transformation can be used instead of the KK transformation.


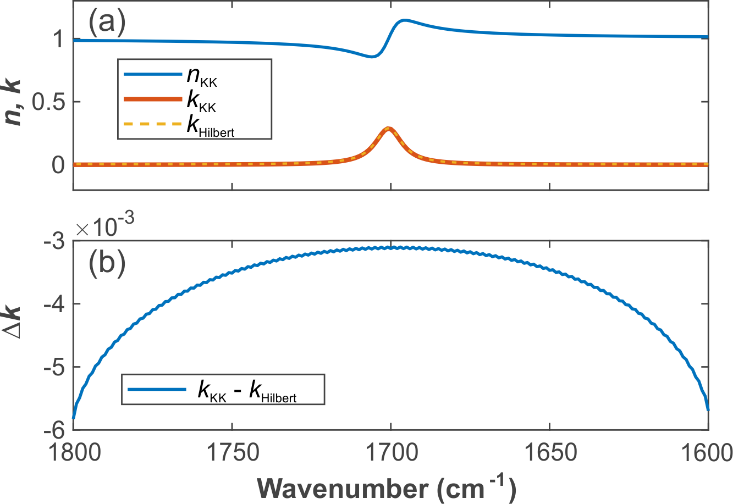


**Figure S1.** Comparison between KK and Hilbert transform in the vicinity of a spectral signature. (a) The $n\left( \text{ν} \right)$ and $k\left( \text{ν} \right)$ from KK and the $k\left( \text{ν} \right)$ calculated by Hilbert transform. (b) The difference $\Delta k\left( \text{ν} \right)$ between the KK and Hilbert curves.

(D)

**Visualization of the Algorithm**


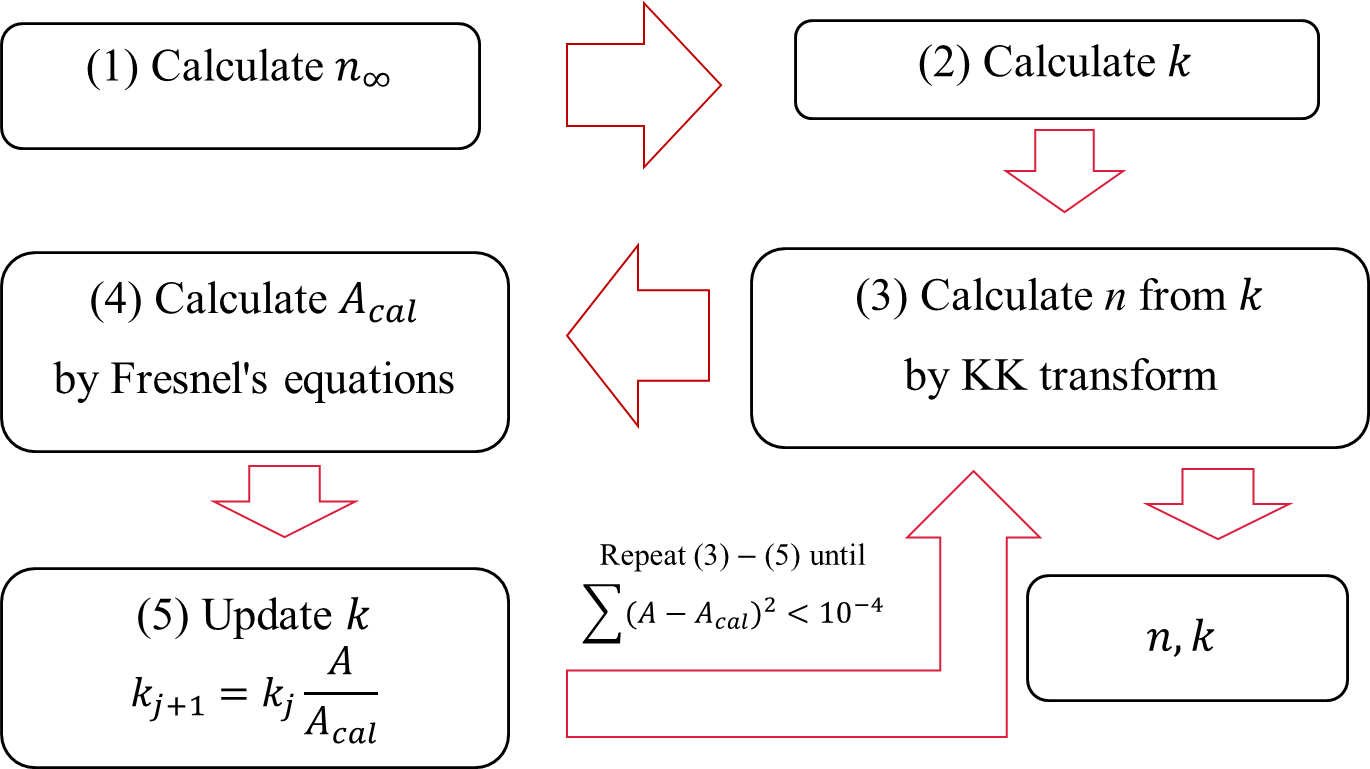


**Figure S2.** Flowchart for calculating the complex refractive index of $n\left( \text{ν} \right)$ and $k\left( \text{ν} \right)$ from the $A\left( \text{ν} \right)$ spectrum. Note that $\hat{n}\left( \text{ν} \right)=n\left( \text{ν} \right)+ik(\text{ν})$.

**Wavelength Dependence of the Refractive Index**

Figure S3 shows the real part of the refractive index $n\left( \text{ν} \right)$ as a function of wavelength. It is worth noting that in the non-absorbing part, the overall trend of $n\left( \text{ν} \right)$ is to decrease with wavenumber (normal dispersion). $n_{\infty}$ is the fraction of the real refractive index that the absorptions in the UV–Vis cause in the limiting case of infinite wavelength (zero wavenumber). If we want to find the refractive index in a specific wavelength or wavenumber range, we must first determine the constant value of the non-absorbing refractive index at infinity. It can be calculated using Eq. 26, and the result is $n_{\infty}$ in specific wavelength or wavenumber range like $n_{\infty IR}$. Eq. 26 describes the overall trend of $n\left( \text{ν} \right)$ without absorbance.


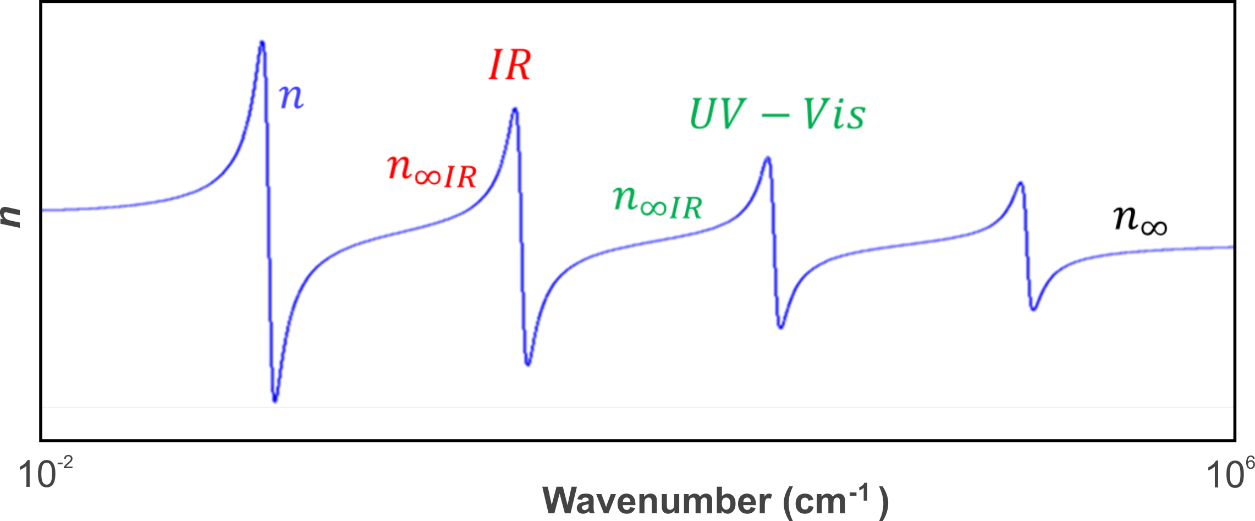


**Figure S3.** The real part of the refractive index varies with wavelength.


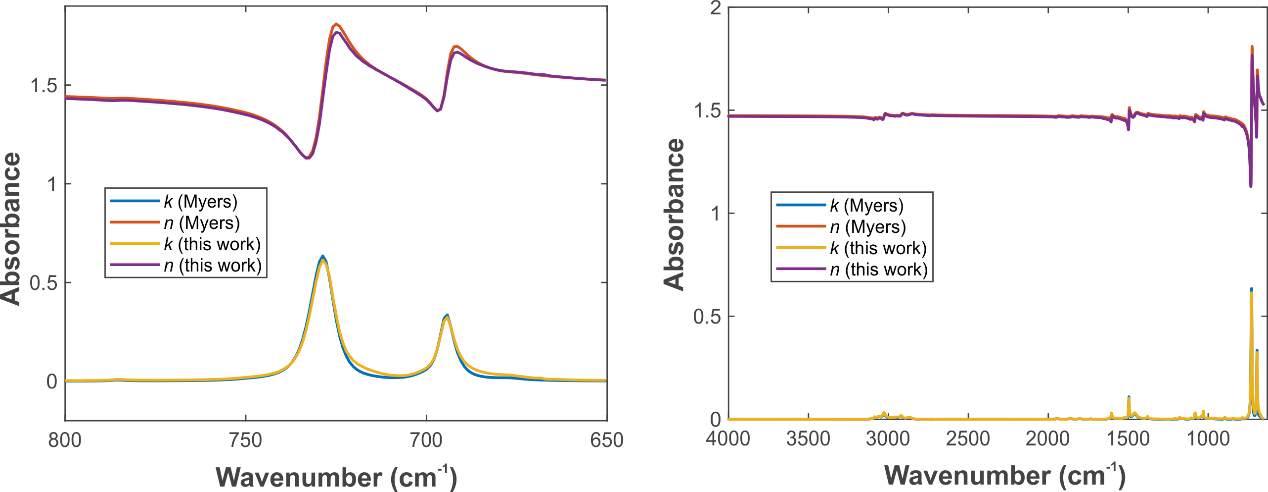


**Figure S4.** The comparison of spectrum between $n\left( \text{ν} \right)$ and $k\left( \text{ν} \right)$ calculated by Myers et al. ^1^ and in this paper.

**Impact of Sample Composition**

Figure S5 shows the differences of the simulated spectrum between Eq. 34 and Eq. 35 for different *x* and *y*. It is worth noting that when we simulate the development trend of the distorted peak, in order to avoid too much interference, we assume that $x_{1}=x_{2}$ ($x_{1}, x_{2}$ are shown in Eq. 36 and Eq. 37, respectively), that is, the two unknowns are merged into $x$. Eq. 35 is the calculation formula for traditional in-phase mixing (usually liquid phase), $x$, usually the volume fraction or mole fraction; in which, $y$ is in Eq. 38.


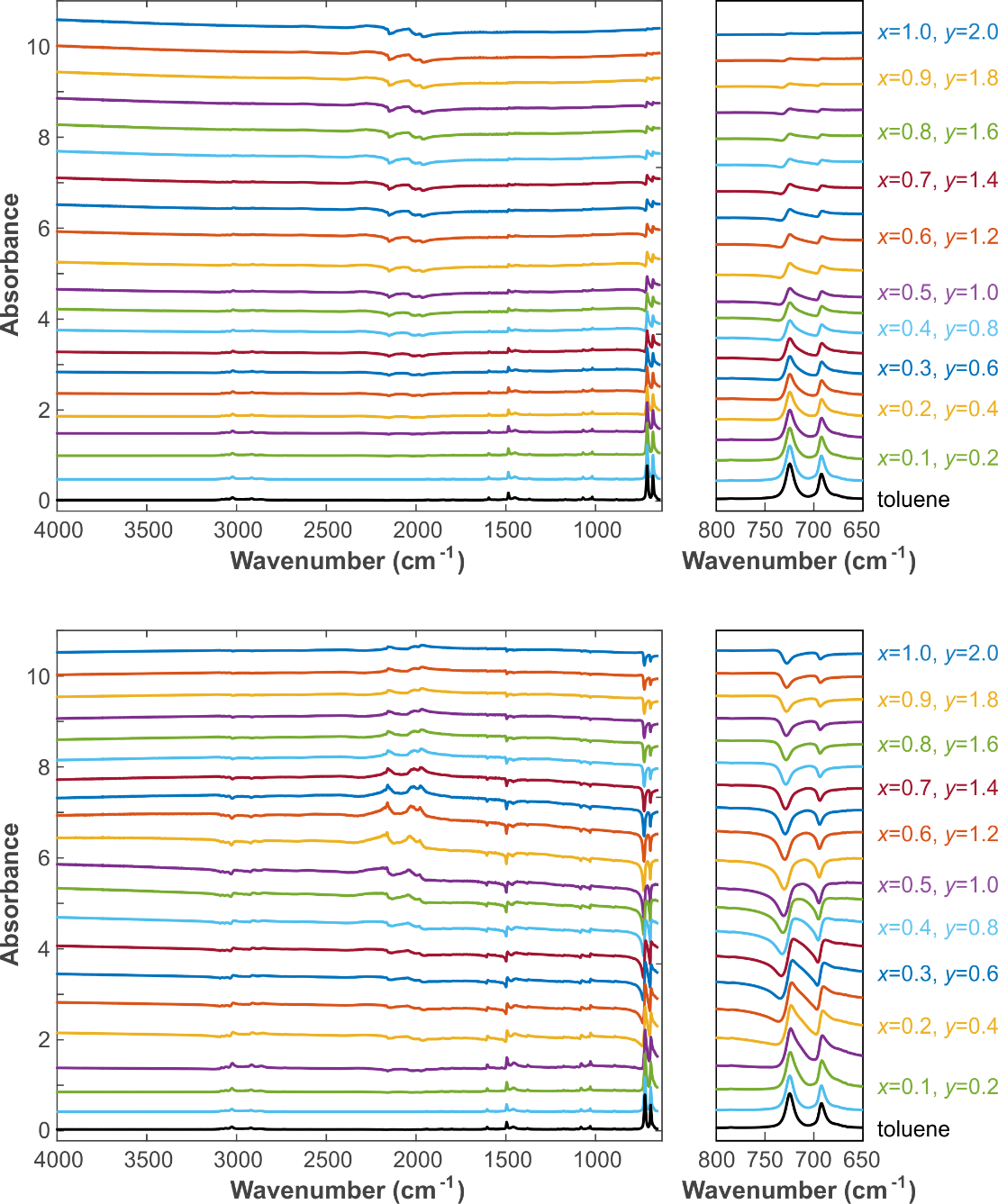


**Figure S5.** The comparison of spectrum between Eq. 34 (upper panel) and Eq. 35 (lower panel) for different *x* and *y*.

It can be clearly seen in Figure S5 that when using Eq. 34, as *x* and *y* increase, the entire spectrum goes through the complete process from normal spectrum to distorted spectrum and then to completely inverted spectrum. When using Eq. 35, as *x* and *y* increase, the entire spectrum eventually almost disappears. This shows that the equation for in-phase mixing cannot be directly applied to solid–liquid mixing.

Liquid-only mixing, on the other hand, tends to be more homogeneous, with interactions primarily occurring at a molecular level without the additional complexities introduced by solid particles. As a result, the spectral behavior and associated trends in liquid-only mixtures can differ significantly from those in solid–liquid systems.
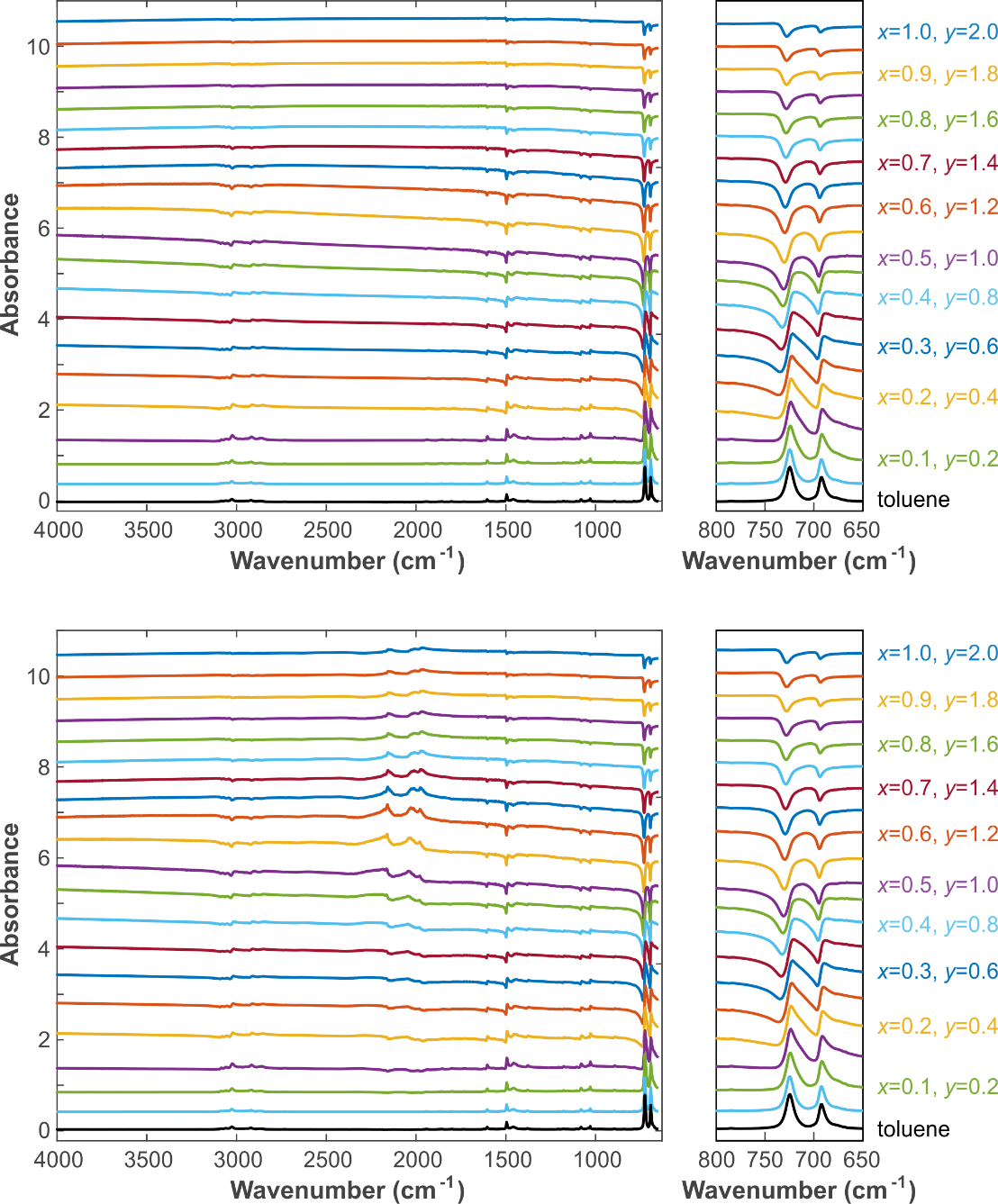


**Figure S6.** Comparison between A (upper panel) and Ab (lower panel) for different *x* and *y*.

In Eq. 38 $A$ refers to the absorption spectrum calculated from the mixture $\hat{n}_{2}\left( \text{ν} \right)$, $A_{22}$ refers to the spectrum of the material with a high refractive index (in our case, carbon black), mainly considering the contribution of carbon black to the baseline shift (because the baseline is basically consistent with the peak of carbon black itself), and $a$ is the constant used for fitting (this is only used when simulating experimental spectra) and is not used in the simulated distortion trend diagram in Figure S6.

**Statistical Data from Fitting**

**Table S1.** Estimated coefficients.

| Estimate SE *t*-Stat *p*-Value | | | | |
| --- | --- | --- | --- | --- |
| $x_{1}$ | −0.017242 | 0.0018319 | −9.4126 | 8.3887e−21 |
| $x_{2}$ | 1.6921 | 0.010039 | 168.56 | 0 |
| $y$ | 1.0793 | 0.0063476 | 170.03 | 0 |
| $a$ | −0.42199 | 0.0050158 | −84.131 | 0 |

Number of observations: 3596, Error degrees of freedom: 3592

Root mean squared error: 0.00485

R-square: 0.999. Adjusted R-square 0.999

F-statistic versus constant model: 8.47e+05, *p*-value = 0

**References**

1. T.L. Myers, R.G. Tonkyn, T.O. Danby, M.S. Taubman et al. “Accurate Measurement of the Optical Constants *n* and *k* for a Series of 57 Inorganic and Organic Liquids for Optical Modeling and Detection”. Appl. Spectrosc. 2018. 72(4): 535–550.
